# In Vivo Prevalence of Beta-Amyloid Pathology and Alzheimer’s Disease Co-Pathology in Idiopathic Normal-Pressure Hydrocephalus—Association with Neuropsychological Features

**DOI:** 10.3390/biomedicines12081898

**Published:** 2024-08-20

**Authors:** Efstratios-Stylianos Pyrgelis, George P. Paraskevas, Vasilios C. Constantinides, Fotini Boufidou, Leonidas Stefanis, Elisabeth Kapaki

**Affiliations:** 1 1st Department of Neurology, School of Medicine, National and Kapodistrian University of Athens, Eginition Hospital, Vass. Sophias Ave. 74, 11528 Athens, Greece; stratospyrg@yahoo.gr (E.-S.P.); vassilis.kon@hotmail.com (V.C.C.); lstefanis@med.uoa.gr (L.S.); 2 Neurochemistry and Biological Markers Unit, 1st Department of Neurology, School of Medicine, National and Kapodistrian University of Athens, Eginition Hospital, Vass. Sophias Ave. 74, 11528 Athens, Greece; geoprskvs44@gmail.com (G.P.P.); fboufidou@med.uoa.gr (F.B.); 3 2nd Department of Neurology, School of Medicine, National and Kapodistrian University of Athens, “Attikon” University General Hospital, Rimini 1, 12462 Athens, Greece

**Keywords:** CSF biomarkers, neuropsychological profile, idiopathic normal-pressure hydrocephalus (iNPH), MMSE, FAB, 5-WT, CLOX-1, CLOX-2, Aβ42, t-Tau, p-Tau

## Abstract

Idiopathic normal-pressure hydrocephalus (iNPH) is a clinic-radiological neurological syndrome presenting with cognitive deficits, gait disturbances and urinary incontinence. It often coexists with Alzheimer’s disease (AD). Due to the reversible nature of iNPH when promptly treated, a lot of studies have focused on possible biomarkers, among which are cerebrospinal fluid (CSF) biomarkers. The aim of the present study was to determine the rate of beta-amyloid pathology and AD co-pathology by measuring AD CSF biomarkers, namely, amyloid beta with 42 and 40 amino acids (Aβ42), the Aβ42/Aβ40 ratio, total Tau protein (t-Tau) and phosphorylated Tau protein at threonine 181 (p-Tau), in a cohort of iNPH patients, as well as to investigate the possible associations among CSF biomarkers and iNPH neuropsychological profiles. Fifty-three patients with iNPH were included in the present study. CSF Aβ42, Aβ40, t-Tau and p-Tau were measured in duplicate with double-sandwich ELISA assays. The neuropsychological evaluation consisted of the Mini-Mental State Examination, Frontal Assessment Battery, Five-Word Test and CLOX drawing tests 1 and 2. After statistical analysis, we found that amyloid pathology and AD co-pathology are rather common in iNPH patients and that higher values of t-Tau and p-Tau CSF levels, as well as the existence of the AD CSF profile, are associated with more severe memory impairment in the study patients. In conclusion, our study has confirmed that amyloid pathology and AD-co-pathology are rather common in iNPH patients and that CSF markers of AD pathology and t-Tau are associated with a worse memory decline in these patients.

## 1. Introduction

Idiopathic normal-pressure hydrocephalus (iNPH) is a communicating form of hydrocephalus, clinically presenting with a triad of symptoms (Hakim’s triad), namely, gait disturbances, cognitive impairment and urinary incontinence [1,2,3]. Brain imaging of iNPH patients either with computed tomography (CT) or with magnetic resonance imaging (MRI) is characterized by ventriculomegaly that cannot be attributed to cortical atrophy, while there are a variety of specific imaging features, mainly narrow sulci in the convexity, the callosal angle, periventricular abnormal densities, the flow void phenomenon in the aqueduct and fourth ventricle, focal bulging of the roof of the lateral ventricles and disproportionally enlarged subarachnoid spaces (DESH) [4,5,6,7]. The exact etiopathogenesis of iNPH is still controversial. Various theories implicate the reduced absorption of cerebrospinal fluid (CSF). Some of them argue that increased venous resistance is the initial “hit”, while others correlate ischemic white matter lesions with the slowing of CSF flow in the extracellular space, resulting in a “counterpressure” effect, which finally leads to the enlargement of the ventricles [8]. There are studies that have shown the disruption of the “glymphatic” system and a consequent delayed clearance in iNPH, which has also been confirmed by studies in animal models [9,10,11]. The common theme in most theories seems to be the fact that iNPH is the result of a vicious circle starting with a disorder of CSF circulation that leads to the deceleration of CSF absorption and the dilation of the brain’s ventricles [3,12].

As recently described for neurodegenerative diseases and other neurological conditions, several circuits seem to play an important role in their pathophysiology [13,14,15,16]. From this perspective, clinical symptoms of iNPH could be partially explained by disturbances in the blood flow and metabolism of periventricular parenchyma. This could lead to the dysfunction of prefrontal pathways and pathways associated with basal ganglia. The aforementioned mechanisms, depicted by both structural and functional imaging studies, are probably associated with both cognitive deficits and gait impairment in iNPH patients [17,18,19]. It has also been suggested that the condensation of specific areas of the midbrain might play a role in gait difficulties [20], while dysfunction of the entorhinal–hippocampal circuit has been associated with cognitive impairment in iNPH patients [21]. The default mode network’s functional connectivity has been found to be decreased in NPH, as it is in AD, and has been associated with a poor cognitive prognosis by studies implementing functional brain MRI [22,23].

Due to the fact that iNPH is potentially reversible with proper management, its timely and accurate diagnosis is of utmost importance. To this end, a lot of studies have revealed the role of specific neuroimaging markers, as mentioned above, regarding the diagnosis, differential diagnosis and, potentially, prognosis of this syndrome [4,6,7]. These markers, however, lack molecular specificity for AD. The prevalence of iNPH increases with age, and it is most common in patients older than 65 years old [24]. Because of this fact, Alzheimer’s disease (AD) comorbidity is relatively common and, according to pathological studies, can reach up to 65% [25,26]. Recently, the existence of AD biomarkers in NPH patients has been suggested to be a potentially pre-AD situation that could benefit from early intervention and possibly be correlated with specific gene expression [27]. Beta-amyloid pathology can also be an inherent characteristic of iNPH [28,29]. The main theory for this fact is that the downregulation of amyloid precursor protein (APP) in the periventricular parenchyma is possibly due to impaired amyloid metabolism and/or a decreased clearance of extracellular fluid into CSF, possibly because tight sulci over the cortexes of iNPH patients could compromise the convective flow of interstitial fluid, resulting in reduced concentrations of APP-derived proteins in CSF [29,30].

There is an adequate number of studies focusing on CSF biomarkers in iNPH [28,31,32,33,34]. Some studies have shown an association between higher levels of phosphorylated Tau at threonine 181 (p-Tau) and/or lower amyloid-beta 1–42 (Aβ42) levels and worse cognitive decline [35,36]. Beta-amyloid pathology could be, at least partially, attributed to the downregulation of the expression of APP [28,37]. A higher p-Tau level has also been correlated with a poorer cognitive outcome after surgery [38]. There are also studies that have investigated the possible association between CSF biomarkers and neuropsychological profiles in AD and Parkinson’s disease dementia [39,40].

The aim of our study is to determine the rate of beta-amyloid pathology and AD co-pathology by measuring Aβ42 and the Aβ42/Aβ40 ratio, total Tau (t-Tau) protein and p-Tau protein in the CSF in a cohort of iNPH patients, as well as to investigate the possible association between CSF biomarkers and iNPH neuropsychological profiles.

## 2. Materials and Methods

### 2.1. Study Population

The cohort analyzed in the current study consisted of 53 subjects. All patients were prospectively recruited in the period 2019–2021 among patients who were admitted to the 1st Department of Neurology of the National and Kapodistrian University of Athens at Eginition Hospital.

For inclusion in the present study, patients had to fulfill the clinic-radiological criteria of probable or possible iNPH according to the recent Guidelines for Management of Idiopathic Normal-Pressure Hydrocephalus [41,42]. All patients underwent detailed clinical, neuropsychological, biochemical and neuroimaging examinations with magnetic resonance imaging (MRI) of the brain. Patients with a medical history or findings of other neurological or systematic diseases with nervous system involvement or brain injury were excluded. Patients with depression, based on their performance on the Geriatric Depression Scale questionnaire, were also excluded due to the fact that depression could affect the cognitive performance of the patients [43,44,45]. The iNPH grading scale was used as a numerical measure to assess the severity of each of the 3 symptoms of iNPH: cognitive decline, gait disturbances and urinary disturbance. Each of these three symptoms is scored from 0 to 4 and then added up to a total score, with higher scores reflecting more severe symptoms [46].

### 2.2. Neuropsychological Assessment

The neuropsychological assessment included the following tests: the Mini-Mental State Examination (MMSE) for an overall cognitive assessment [47], the Frontal Assessment Battery (FAB) [48] for detecting deficits associated with frontal lobe functions, the 5-word immediate and delayed recall (5WT) [49] for assessing memory deficits, and the 15-point spontaneous and copy CLOX drawing (CLOX1 and 2, respectively) for detecting visuospatial and frontal impairment (CLOX1) (CLOX2) [50]. These tests were selected as they are well established, some of them validated in the Greek population [51], and easy to perform in a bedside setup. The neuropsychological assessments were carried out by the same neurologist with experience in the specific field.

### 2.3. CSF Sampling, Biomarker Analysis and Sub-Group Creation

A lumbar puncture was performed in all patients in the morning hours after all-night fasting using well-established processes according to guidance about the consistency of pre-analytical confounding factors [52]. The opening CSF pressure was assessed, and patients with opening pressure greater than 200 mm H_2_O were excluded. CSF samples were then collected in appropriate polypropylene tubes. Then, the samples were centrifugated, split into aliquots (of 0.5 mL) and then frozen (at −80 °C). Each aliquot was defrosted once, and then it went through analysis. CSF biomarkers (Aβ42, Aβ40, t-Tau and p-Tau) were measured in duplicate with ELISA by commercially available kits from EUROIMMUN, applied in the fully automated analyzer EUROIMMUN Analyzer I (EUROIMMUN, Medizinische Labordiagnostika AG, Lübeck, Germany). The Aβ42/Aβ40 ratio was then calculated.

Patients were then divided into amyloid-positive, based on decreased values of Aβ42 and/or Aβ42/Aβ40, and amyloid-negative; t-Tau-positive and t-Tau-negative; p-Tau-positive and p-Tau-negative; and patients with an AD CSF biomarker profile and patients with a non-AD CSF biomarker profile according to our laboratory cut-off values, with sensitivity and specificity over 80%, as described elsewhere [53]. Finally, patients with increased p-Tau, decreased Aβ42 and/or Aβ42/Aβ40 and increased t-Tau or neurodegeneration revealed in neuroimaging (brain MRI) were considered to have underlying AD co-pathology according to the NIA-AA research group and were classified into the sub-group of patients with an AD CSF profile [54,55]. All the rest were considered to have a non-AD CSF profile.

### 2.4. Statistical Analysis

All numerical parameters were tested for normality of distribution and homogeneity of variances by the Shapiro–Wilk and Brown–Forsyth tests, respectively. Regarding variables without normal distributions and homogeneous variances, nonparametric tests were used for statistical analysis. The Mann–Whitney U Test was implemented to look into differences in the median values of MMSE, FAB, 5-WT immediate and delayed recall, and CLOX-1 and CLOX-2 among amyloid-positive and amyloid-negative, t-Tau-positive and t-Tau-negative, p-Tau-positive and p-Tau-negative, and AD- and non-AD-profiled iNPH patients. Categorical data were compared between groups using the χ2-test. All analyses were carried out using IBM SPSS Statistics^®^ version 23.0.0.0 (SPSS Inc., Chicago, IL, USA, 2013). In all tests, a *p*-value of 5% or lower was considered statistically significant, and interval estimators are reported with 95% confidence. All graphs were designed using GraphPad Prism^®^, version 8.43 (GraphPad Software Inc., La Jolla, CA, USA, 2020).

### 2.5. Ethical Aspects

The study was performed in accordance with the ethical principles of the Declaration of Helsinki (1964) and had the approval of the Scientific and Ethics Committee of our center. All patients and/or their closest relatives provided written informed consent for involvement in the study. Patients’ data have undergone anonymization and have been treated with respect and confidentiality. 

## 3. Results

A total of 53 patients participated in this study, whose demographic data are summarized in Table 1. 

**Figure 1:**
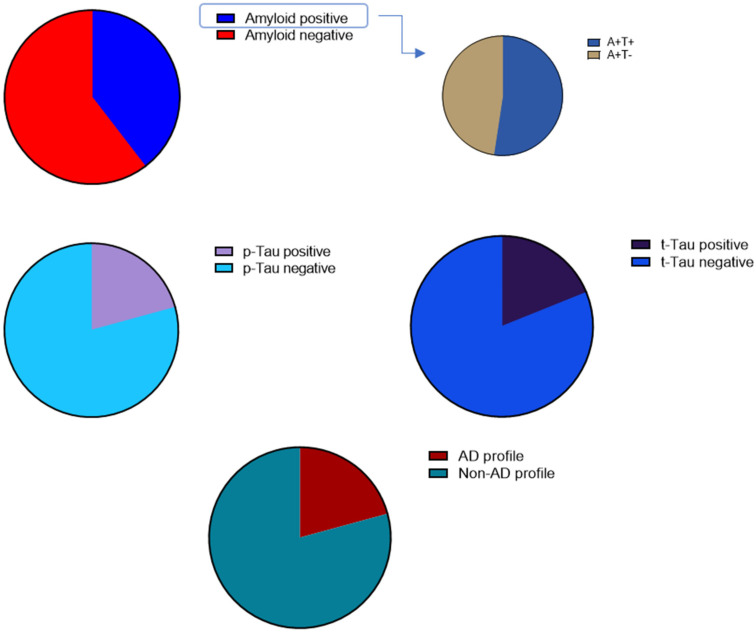
Fifty-three iNPH patients who, based on their CSF biomarker values, were divided into “amyloid-positive” (A+T+ and A+T−) and “amyloid-negative”, “t-Tau-positive” and “t-Tau-negative”, “p-Tau-positive” and “p-Tau-negative” and patients with AD and non-AD profiles.

Patients were then divided, based on their CSF biomarkers’ values, into amyloid-positive and amyloid-negative, t-Tau-positive and t-Tau-negative, p-Tau-positive and p-Tau-negative (according to our laboratory cut-off values) and patients with AD and non-AD profiles (Figure 1). Based on the proposal of the NIA-AA research group for a biological definition of AD, only A+T+ patients were classified as having an AD profile [36] (Figure 1).

There were 21 patients classified as amyloid-positive, with 20 of them having decreased values of both Aβ42 and the Aβ42/Aβ40 ratio and 1 of them having normal Aβ42 value but a decreased Aβ42/Aβ40 ratio.

Disease duration, age and years of education did not differ significantly among the aforementioned sub-groups of patients. The neuropsychological characteristics of each of the aforementioned sub-groups are depicted in Table 2, Table 3, Table 4 and Table 5.

After post hoc analysis (with Bonferroni correction), no statistically significant differences remained.

**Figure 2:**
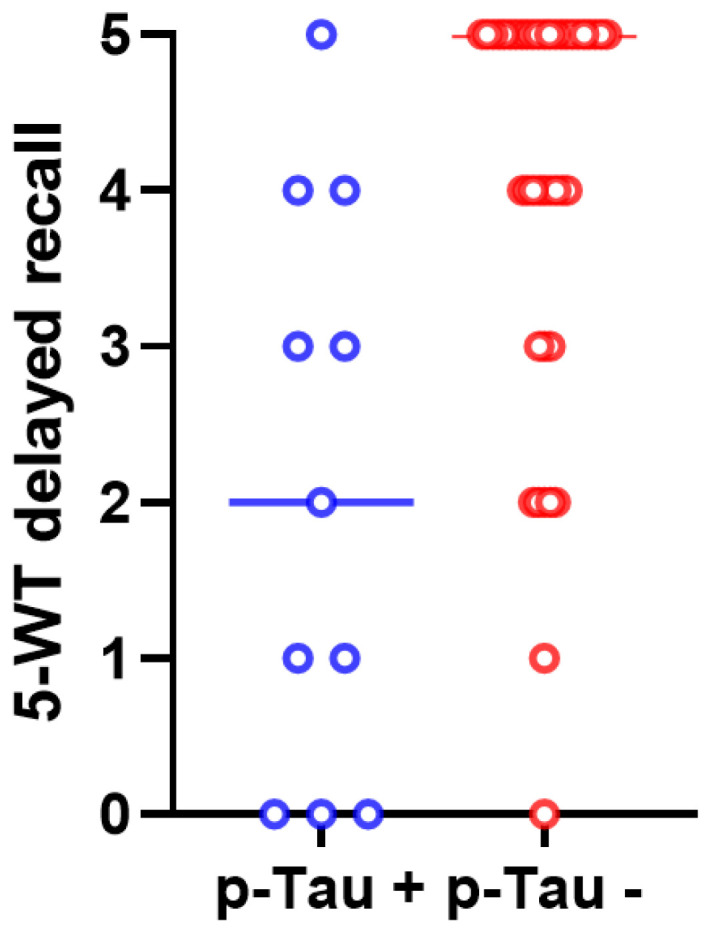
The scores of delayed recall on the 5-WT differed significantly between p-Tau-positive (in blue color) and p-Tau-negative (in red color) iNPH patients (*p* = 0.003). The median values and the ranges of these values in the two groups are presented in this graph.

After post hoc analysis (with Bonferroni correction), the difference in delayed recall on the 5-WT between the p-Tau-positive and p-Tau-negative patients remained statistically significant (Figure 2).

**Figure 3:**
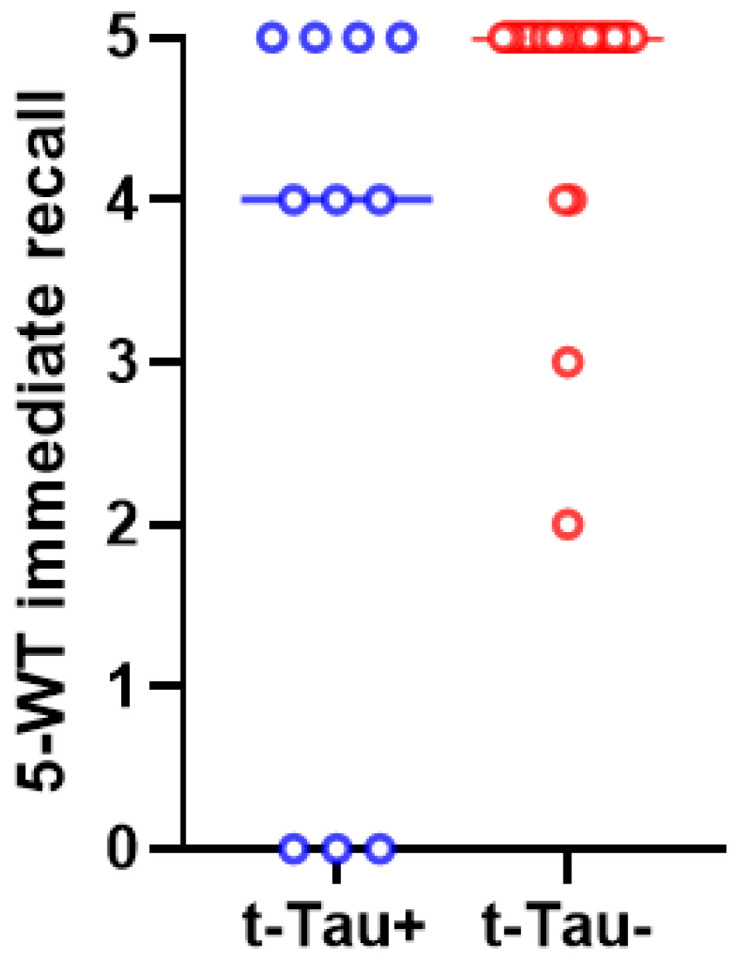
The scores of 5-WT immediate recall differed significantly between t-Tau-positive (in blue color) and t-Tau-negative (in red color) iNPH patients (*p* = 0.005). The median values and the ranges of these values in the two groups are presented in this graph.

**Figure 4:**
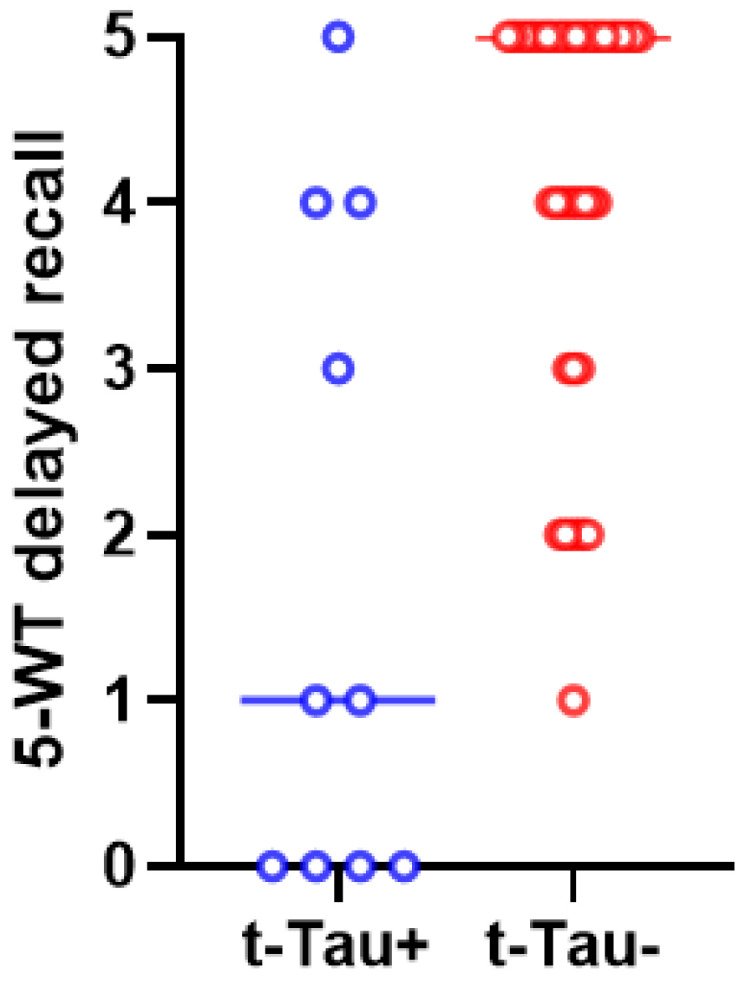
The scores of 5-WT delayed recall differed significantly between t-Tau-positive (in blue color) and t-Tau-negative (in red color) iNPH patients (*p* = 0.001). The median values and the ranges of these values in the two groups are presented in this graph.

After post hoc analysis (with Bonferroni correction), only the differences in immediate and delayed recall on the 5-WT between the t-Tau-positive and t-Tau-negative patients remained statistically significant (Figure 3 and Figure 4).

Out of 21 amyloid-positive patients, 11 had an A+T+ profile and 10 had an A+T−. As mentioned above, based on the proposal of the NIA-AA research group for a biological definition of AD, only A+T+ patients were classified as having an AD profile [36].

**Figure 5:**
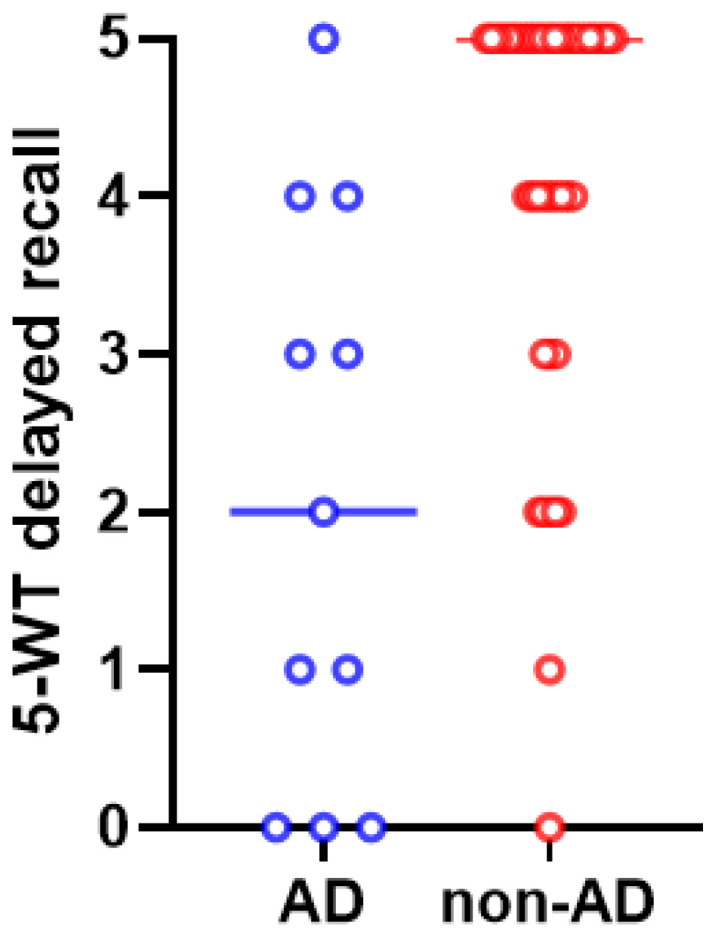
The scores of 5-WT delayed recall differed significantly between iNPH patients with AD (in blue color) and non-AD (in red color) CSF profiles (*p* = 0.003). The median values and the ranges of these values in the two groups are presented in this graph.

After post hoc analysis (with Bonferroni correction), the difference in delayed recall on the 5-WT between patients with AD and non-AD CSF profiles remains statistically significant (Figure 5).

## 4. Discussion

In the present study, we aimed to associate CSF biomarker positivity and the total profile with specific patterns of neuropsychological deficits in a cohort consisting of iNPH patients.

Regarding amyloid positivity, we chose to include patients with either decreased Aβ42 and/or a decreased Aβ42/Aβ40 ratio, which may be superior to Aβ42 alone, as has been previously described by our group and others [53,56]. Another finding of this study is that all patients with an AD profile had abnormal Aβ42/Aβ40 ratios, possibly suggesting that Aβ42/Aβ40 could assist in differentiating AD from iNPH. This seems to be in accordance with the findings of Kim et al. (2019) that the Aβ42/Aβ40 ratio is significantly lower in AD than in iNPH patients and healthy individuals [57]. The results of previous studies have suggested that lower Aβ42 CSF concentrations are related to more severe cognitive impairment [58]. In our study, amyloid-positive patients demonstrated lower scores in delayed recall on the 5-WT than amyloid-negative patients, but the association was not strong enough and disappeared after Bonferroni correction.

Our study shows that p-Tau-positive patients have worse performance in delayed recall on the 5-WT than p-Tau-negative patients. In other studies, high CSF p-Tau levels have also been found to correlate with a worse cognitive prognosis [38,59]. Regarding a possible association between t-Tau levels and cognitive performance, our data also show that iNPH patients with higher t-Tau CSF levels tend to have lower scores in immediate and delayed recall on the 5-WT. Other studies had also concluded that high t-Tau levels are related to worse iNPH clinical symptoms [31,35].

The AD CSF profile in iNPH patients seems to correlate with a cognitive impairment of the amnestic subtype in contrast to a non-AD profile, which is associated with better neurocognitive performance. A study by Golomb et al. (2000) suggested that coexisting AD pathology in iNPH patients does not notably alter the response to shunt surgery [25], while Lim et al. in 2014 found that concomitant AD pathology in these patients might lead to non-responsiveness either to a Tap-test or to shunting [36]. Müller-Schmitz et al. (2020) concluded that iNPH patients with AD comorbidity significantly improved kinetically and neuropsychologically after CSF removal, while patients with only iNPH did not [60]. On the contrary, a recent meta-analysis concluded that the CSF levels of t-Tau and p-Tau are significantly higher among iNPH patients whose symptoms did not improve after shunt placement compared to those whose symptoms improved [61]. Subsequently, CSF biomarkers may not be the main factor in iNPH diagnosis, as clinical and radiological features play key roles; however, they are of value in the differential diagnosis and potentially the prognosis of iNPH patients with concomitant AD pathology, who may be less responsive to shunt placement [36,61]. Thus, the findings of the present study suggest that the results of CSF biomarkers may be taken into account regarding shunt responsiveness.

To summarize, rather common beta-amyloid pathology and AD co-pathology, as well as worse cognitive performance related to increased CSF levels of t-Tau and p-Tau, seem to be in accordance with the findings of other studies, as analyzed above. The present study has certain limitations. First, there is a lack of neuropathological evidence of iNPH; however, this is an inherent disadvantage of relevant studies due to the fact that there are no solid pathological findings in iNPH. The number of patients included is rather small, in accordance with single-center studies; notably, such studies are more homogeneous and avoid inter-rater variability, especially regarding neuropsychological testing. Our purpose was to use this study as a pilot study in order to draw some preliminary conclusions so we can implement the same methodology on larger cohorts in the future. Another limitation could be that, despite having excluded other major medical conditions, some parameters like smoking or medication could have played a confounding factor role in the present study. Nevertheless, such factors remained stable before and after the LP, diminishing that possibility. Additionally, due to the cross-sectional design of the current study, longitudinal data about the correlations between CSF biomarkers and neuropsychological performance are, unfortunately, unavailable. It is worth noting that simultaneous neuropsychological and neurochemical assessments of a clinically well-characterized cohort of iNPH patients took place in our study in an attempt to reveal possible CSF biomarker-specific neuropsychological deficits/profiles.

Further studies performing analyses on larger samples are necessary in order to verify the association between biomarker profiles and neuropsychological performance and to evaluate the potential predictive role of these biomarkers through long-term follow-up.

## 5. Conclusions

In conclusion, our study has confirmed that amyloid pathology is rather common in iNPH patients. It has also given supporting evidence that CSF markers of AD pathology and t-Tau (a non-specific neurodegeneration marker) are associated with worse memory decline in these patients. 

Our results show that AD co-pathology in iNPH patients is associated with worse performance on neuropsychological tests regarding memory. Further studies are required to investigate whether the comorbidity of neurodegenerative diseases like AD could play a de novo prognostic role for iNPH patients.

## Figures and Tables

**Table 1:** Demographic data of patients.

Gender	22 Females/31 Male Patients
Age	75 (69.5–77)
Years of education	12 (6–16)
Disease duration	24 (13–48)
iNPH grading scale	6 (5–7)

Numerical data are presented as median values (25th–75th percentile).

**Table 2:** Neuropsychological data of amyloid-positive and amyloid-negative patients.

Neuropsychological Test	Amyloid-Positive Patients Ν = 21	Amyloid-Negative Patients Ν = 32	*p*
MMSE	22 (17.5–26)	23.5 (18.25–28)	0.3167 ^†^
FAB	10 (6–13.5)	12 (9–13)	0.3664 ^†^
5-WT immediate recall	5 (4.5–5)	5 (5–5)	0.6794 ^†^
5-WT delayed recall	4 (1.5–5)	5 (4–5)	0.0145 ^†^*
CLOX-1	6 (2–11)	8 (5–11)	0.2083 ^†^
CLOX-2	10 (4.5–13.5)	11.5 (9–13)	0.5162 ^†^

N: number of patients; MMSE: Mini-Mental State Examination; FAB: Frontal Assessment Battery; 5-WT: 5-word test; CLOX-1 and 2: 15-point spontaneous and copy CLOX drawing, respectively. Neuropsychological data are presented as median values (25th–75th percentile). ^†^ Mann–Whitney U test; statistically significant *p* values are noted with *.

**Table 3:** Neuropsychological data of p-Tau-positive and p-Tau-negative patients.

Neuropsychological Test	p-Tau-Positive Patients Ν = 11	p-Tau-Negative Patients Ν = 42	*p*
MMSE	23 (11–29)	24 (18.75–27)	0.252 ^†^
FAB	11 (5–14)	11.5 (9–13)	0.640 ^†^
5-WT immediate recall	5 (4–5)	5 (5–5)	0.1326 ^†^
5-WT delayed recall	2 (0–4)	5 (3.75–5)	0.0004 ^†^*
CLOX-1	5 (1–13)	8 (5–11)	0.6446 ^†^
CLOX-2	11 (1–14)	11 (8–13)	0.7649 ^†^

N: number of patients; MMSE: Mini-Mental State Examination; FAB: Frontal Assessment Battery; 5-WT: 5-word test; CLOX-1 and 2: 15-point spontaneous and copy CLOX drawing, respectively. Neuropsychological data are presented as median values (25th–75th percentile). ^†^ Mann–Whitney U test; statistically significant *p* values are noted with *.

**Table 4:** Neuropsychological data of t-Tau-positive and t-Tau-negative patients.

Neuropsychological Test	t-Tau-Positive Patients Ν = 10	t-Tau-Negative Patients Ν = 43	*p*
MMSE	17.5 (11–23)	24 (20–28)	0.0128 ^†^*
FAB	8 (2.5–12)	12 (9–13)	0.0477 ^†^*
5-WT immediate recall	4 (0–5)	5 (5–5)	0.0008 ^†^*
5-WT delayed recall	1 (0–4)	5 (4–5)	0.0002 ^†^*
CLOX-1	4.5 (0.75–7.75)	8 (5–11)	0.036 ^†^*
CLOX-2	5 (0–12.5)	12 (9–13)	0.0285 ^†^*

N: number of patients; MMSE: Mini-Mental State Examination; FAB: Frontal Assessment Battery; 5-WT: 5-word test; CLOX-1 and 2: 15-point spontaneous and copy CLOX drawing, respectively. Neuropsychological data are presented as median values (25th–75th percentile). ^†^ Mann–Whitney U test; statistically significant *p* values are noted with *.

**Table 5:** Neuropsychological data of patients with AD and non-AD profile.

Neuropsychological Test	Patients with AD Profile Ν = 11	Patients with Non-AD Profile Ν = 42	*p*
MMSE	23 (11–29)	24 (18.75–27)	0.252 ^†^
FAB	11 (5–14)	11.5 (9–13)	0.640 ^†^
5-WT immediate recall	5 (4–5)	5 (5–5)	0.1326 ^†^
5-WT delayed recall	2 (0–4)	5 (3.75–5)	0.0004 ^†^*
CLOX-1	5 (1–13)	8 (5–11)	0.6446 ^†^
CLOX-2	11 (1–14)	11 (8–13)	0.7649 ^†^

N: number of patients; MMSE: Mini-Mental State Examination; FAB: Frontal Assessment Battery; 5-WT: 5-word test; CLOX-1 and 2: 15-point spontaneous and copy CLOX drawing, respectively. Neuropsychological data are presented as median values (25th–75th percentile). ^†^ Mann–Whitney U test; statistically significant *p* values are noted with *.

## Data Availability

The data presented in this study are available upon reasonable request from the corresponding author. The data are not publicly available due to privacy restrictions.
